# Diagnostic Accuracy of Smartwatches for the Detection of Cardiac Arrhythmia: Systematic Review and Meta-analysis

**DOI:** 10.2196/28974

**Published:** 2021-08-27

**Authors:** Scarlet Nazarian, Kyle Lam, Ara Darzi, Hutan Ashrafian

**Affiliations:** 1 Imperial College London London United Kingdom

**Keywords:** wearables, smartwatch, cardiac arrhythmia, atrial fibrillation, cardiology, mHealth, wearable devices, screening, diagnostics, accuracy

## Abstract

**Background:**

Significant morbidity, mortality, and financial burden are associated with cardiac rhythm abnormalities. Conventional investigative tools are often unsuccessful in detecting cardiac arrhythmias because of their episodic nature. Smartwatches have gained popularity in recent years as a health tool for the detection of cardiac rhythms.

**Objective:**

This study aims to systematically review and meta-analyze the diagnostic accuracy of smartwatches in the detection of cardiac arrhythmias.

**Methods:**

A systematic literature search of the Embase, MEDLINE, and Cochrane Library databases was performed in accordance with the PRISMA (Preferred Reporting Items for Systematic Reviews and Meta-Analyses) guidelines to identify studies reporting the use of a smartwatch for the detection of cardiac arrhythmia. Summary estimates of sensitivity, specificity, and area under the curve were attempted using a bivariate model for the diagnostic meta-analysis. Studies were examined for quality using the Quality Assessment of Diagnostic Accuracy Studies 2 tool.

**Results:**

A total of 18 studies examining atrial fibrillation detection, bradyarrhythmias and tachyarrhythmias, and premature contractions were analyzed, measuring diagnostic accuracy in 424,371 subjects in total. The signals analyzed by smartwatches were based on photoplethysmography. The overall sensitivity, specificity, and accuracy of smartwatches for detecting cardiac arrhythmias were 100% (95% CI 0.99-1.00), 95% (95% CI 0.93-0.97), and 97% (95% CI 0.96-0.99), respectively. The pooled positive predictive value and negative predictive value for detecting cardiac arrhythmias were 85% (95% CI 0.79-0.90) and 100% (95% CI 1.0-1.0), respectively.

**Conclusions:**

This review demonstrates the evolving field of digital disease detection. The current diagnostic accuracy of smartwatch technology for the detection of cardiac arrhythmias is high. Although the innovative drive of digital devices in health care will continue to gain momentum toward screening, the process of accurate evidence accrual and regulatory standards ready to accept their introduction is strongly needed.

**Trial Registration:**

PROSPERO International Prospective Register of Systematic Reviews CRD42020213237; https://www.crd.york.ac.uk/prospero/display_record.php?RecordID=213237.

## Introduction

### Background

Cardiac arrhythmia encompasses a group of conditions in which the heart beats too quickly, too slowly, or in an irregular pattern. Significant morbidity, mortality, and financial burden are associated with cardiac rhythm abnormalities [1]. Of these cardiac rhythm abnormalities, atrial fibrillation (AF) is the most common type of cardiac arrhythmia [2], and its prevalence increases sharply with age, reaching 17.8% in a European population for those aged >85 years [3,4]. The presence of AF increases the risk of ischemic stroke by five-fold [5] and can lead to other thromboembolic events. It is well recognized that AF often remains asymptomatic, and therefore, by the time of screening, the patient may have already suffered the consequences.

Although AF is the most common type of cardiac arrhythmia, other arrhythmias, such as premature cardiac contractions, are responsible for significant symptomatic burden. Premature atrial contractions have been shown to be an independent risk factor for all strokes in a longitudinal study [6]. Similarly, a cohort study found that having premature ventricular contractions resulted in a higher rate of ischemic stroke than those without contractions [7].

Conventional screening tools, in the form of 12-lead electrocardiograms (ECGs) and ambulatory electrocardiography monitors, are often unsuccessful in detecting AF or other cardiac arrhythmias, such as bradyarrhythmias or tachyarrhythmias, because of the transient nature of episodes. The episodic and infrequent nature of cardiac arrhythmias means that they are not captured within the investigation period, making diagnosis very difficult.

Recent advances in mobile health technology and wearable electronic devices allow heart rhythm monitoring to be undertaken in real time with greater comfort, ease, and engagement [8]. Wearable devices such as smartwatches show great potential for the detection of cardiac arrhythmias. Timely diagnosis of AF ensures that management is commenced early to prevent ensuing events that impact the quality of life while also relieving the burden that this poses on the health care system.

Smartwatches have gained popularity in recent years, especially as a health tool for the detection of heart rhythms. Patients with a smartwatch can self-diagnose their heart rhythm within 30 seconds using one finger [9]. These apps use photoplethysmography (PPG) from an optical sensor to analyze the pulse rate from the wrist [10]. However, adoption of the technology by both clinicians and patients requires that these devices are accurate and provide clinically applicable information in a manner that is compatible with workflow in the health setting.

### Objectives

This study aims to systematically review and meta-analyze the diagnostic accuracy of smartwatches in the detection of cardiac arrhythmias.

## Methods

### Overview

This review was carried out and reported in accordance with the PRISMA (Preferred Reporting Items for Systematic Reviews and Meta-Analyses) statement [11]. The review was registered at the International Prospective Register of Systematic Reviews (PROSPERO ID: CRD42020213237).

### Search Strategy

A thorough literature search was performed using the Embase, MEDLINE, and Cochrane Library databases. All articles published until February 2021 were included in the study. The appropriate MeSH (Medical Subject Headings) terms and free text all field searches were performed and combined with appropriate Boolean operator terms for arrhythmias, cardiac OR irregular pulse* OR atrial fibrillation, wearable electronic devices OR smartwatch* OR wristband*, diagnosis, computer-assisted OR diagnos*, and detect* in Embase and Ovid in MEDLINE. Search terms in the Cochrane Library included arrhythmias, cardiac OR atrial fibrillation OR irregular pulse* OR arrhythmia*, smartwatch* OR wearable electronic device*, and diagnosis, computer-assisted OR detect* OR diagnos*. The full search strategy is provided in Multimedia Appendix 1.

### Inclusion and Exclusion Criteria

Inclusion criteria were as follows:

- studies reporting detection of cardiac arrhythmias using smartwatches;
- studies reporting sensitivity, specificity and diagnostic accuracy; or studies with adequate information to calculate these data; and
- studies published or translated into English.

Exclusion criteria were as follows:

- studies with no original data present (eg, review article, letter);
- studies with no full text available;
- studies >20 years; and
- studies without adequate data to calculate sensitivity, specificity and diagnostic accuracy data.

### Study Selection

Studies obtained from the literature search were analyzed, and duplicates were removed. Title, abstract, and full-text review were performed by 2 reviewers independently, and irrelevant studies were excluded. Disagreements were settled by consensus among the reviewers.

### Data Extraction

Data were extracted onto a standard spreadsheet template. Information regarding the journal, author, study design, type of smartwatch, number of subjects, and diagnostic accuracy data (sensitivity, specificity, accuracy, positive predictive value [PPV], and negative predictive value [NPV]) was selected from each paper.

### Study Quality Assessment

The Quality Assessment of Diagnostic Accuracy Studies 2 tool was used to assess the risk of bias of the included studies [12]. Each domain was classified as low risk, high risk, or unclear risk of bias.

### Statistical Analysis

Summary estimates of sensitivity, specificity, and area under the curve data were attempted using a bivariate model for diagnostic meta-analysis. Independent proportions and their differences were calculated and pooled using DerSimonian and Laird random effects modeling [13]. This considered both between-study and within-study variances, which contributed to study weighting. Study-specific estimates and 95% CIs were computed and represented in forest plots. Statistical heterogeneity was determined by the *I^2^* statistic, where <30% was low, 30%-60% was moderate, and >60% was high. Analyses were performed using Stata version 15 (StataCorp). *P* values of ≤.05 were considered statistically significant.

## Results

### Search Results and Characteristics

**Figure 1:**
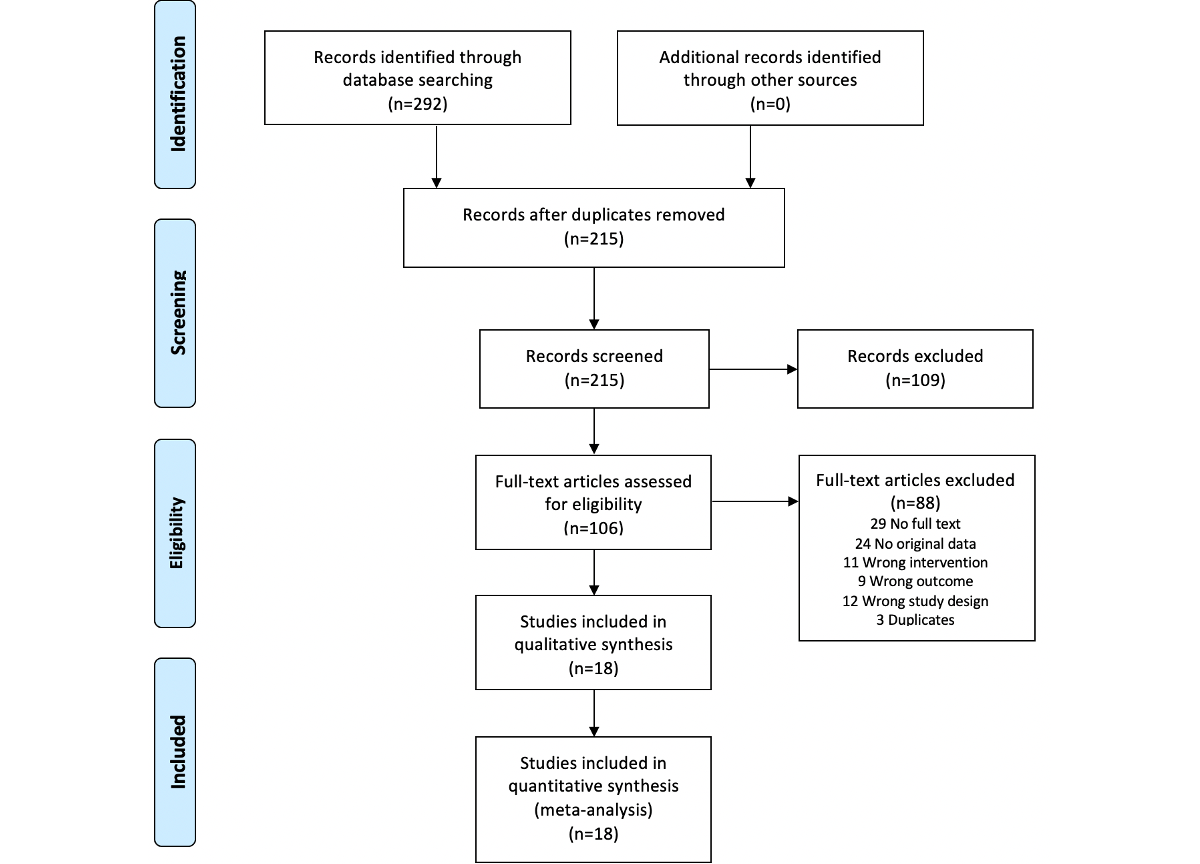
PRISMA (Preferred Reporting Items for Systematic Reviews and Meta-Analyses) flow diagram for study selection.

The database searches identified 292 studies that matched the criteria. Duplicates were removed, and 215 studies were eligible for title and abstract screening. Following this, a full-text review was undertaken, and a total of 18 studies were included in this review. Studies that failed to satisfy the inclusion criteria were excluded, and the reasons for exclusion of these articles included wrong intervention (such as the lack of use of a smartwatch) or wrong outcomes (such as studies that did not involve the detection of cardiac arrhythmias or reports on diagnostic accuracy). The study screening and selection process is shown in Figure 1.

The studies included in this systematic review were all published between 2017 and 2021. The outcome measure in the studies was mainly AF detection but also included bradyarrhythmias, tachyarrhythmias, and premature contractions. The studies measured diagnostic accuracy using smartwatches in 424,371 subjects in total. The Apple watch was used in 7 studies, Samsung smartwatches were used in 5 studies, and the remaining studies used a Huawei, Huami, or Empatica smartwatch. One study used the Wavelet wristband. Three different types of Huawei smartwatches were used in 2 studies to assess the diagnostic accuracy [14,15].

The reference standard was an ECG in most studies in the form of a 12-lead ECG, a Holter monitor, an ECG patch, telemetry, or an internet-enabled mobile ECG. In one study, an implantable cardiac monitor was used as the standard [16]. Almost all studies, except for 2 that did not specify, used PPG-based sensors to assess pulse rate. Table 1 provides the characteristics of the included studies.

**Table 1:** Characteristics of included studies on detection of cardiac arrhythmias.

Authors (year)	Primary outcome	Study design	Type of sensor	Reference standard	Research or real-life setting	Type of smartwatch	Number of subjects
Corino et al (2017) [17]	AF^a^ detection	Prospective	PPG^b^	—^c^	Research	Empatica E4	70
Bumgarner et al (2018) [18]	AF detection	Prospective, nonrandomized, adjudicator blinded	—	12-lead ECG^d^ (physician reviewed)	Research	Apple watch	100
Tison et al (2018) [19]	AF detection	Multinational, cohort	PPG	12-lead ECG	Research	Apple watch	1617
Wasserlauf et al (2019) [16]	AF detection	Prospective	PPG	Insertable cardiac monitor	Research	Apple watch	24
Perez et al (2019) [20]	AF detection	Prospective, single group, open label, site less, pragmatic	PPG	ECG patch	Real life	Apple watch	419,297
Zhang et al (2019) [14]	AF detection	Pilot, cohort	PPG	12-lead ECG and physical examination	Real life	Huawei Watch GT	263
						The Honor Watch (Huawei)	263
						The Honor Band4 (Huawei)	209
Ding et al (2019) [21]	AF detection	Observational	PPG	Holter monitor ECG	Research	Samsung Simband 2	40
Dorr et al (2019) [22]	AF detection	Prospective, two center, case-control	PPG	Internet-enabled mobile ECG	Research	Samsung GearFit 2	508
Bashar et al (2019) [23]	AF detection	Prospective	PPG	Holter monitor ECG	Research	Samsung Simband	20
Bashar et al (2019) [24]	AF detection	Prospective	PPG	Holter monitor ECG	Research	Samsung Simband	37
Valiaho et al (2019) [25]	AF detection	Multicenter prospective case-control	PPG	Three-lead ECG	Research	Empatica E4	213
Guo et al (2019) [15]	AF detection	Prospective	PPG	Clinical evaluation, ECG, or 24-hour Holter monitoring	Real life	Huawei Watch GT	212
						The Honor Watch (Huawei)	265
						The Honor Band4 (Huawei)	264
Chen et al (2020) [26]	AF detection	Prospective	PPG	12-lead ECG (physician reviewed)	Research	Amazfit Health Band 1S (Huami)	401
Rajakariar et al (2020) [27]	AF detection	Prospective, multicenter validation	PPG	12-lead ECG	Research	Apple watch	200
Seshadri et al (2020) [28]	AF detection	Prospective	—	Telemetry	Research	Apple watch	50
Selder et al (2020) [29]	AF detection	Observational, prospective cohort	PPG	One-lead ECG	Research	Wavelet wristband	60
Han et al (2020) [30]	Premature atrial contraction or premature ventricular contraction	Prospective	PPG	ECG patch	Research	Samsung Gear S3	2
Caillol et al (2021) [31]	AF, atrial flutter, brady arrhythmias, and tachyarrhythmias	Prospective	PPG	12-lead ECG	Research	Apple watch	256

^a^AF: atrial fibrillation.

^b^PPG: photoplethysmography.

^c^Not available.

^d^ECG: electrocardiogram.

### Sensitivity, Specificity, and Accuracy

**Figure 2:**
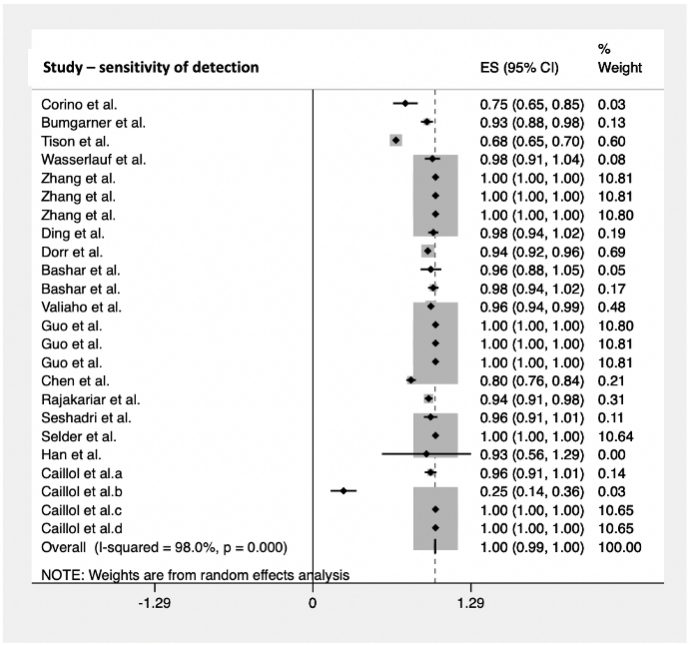
Pooled analysis for sensitivity of cardiac arrhythmia detection by smartwatches. Effect sizes are shown with 95% CIs. A random effects model was used. ES: effect sizes.

**Figure 3:**
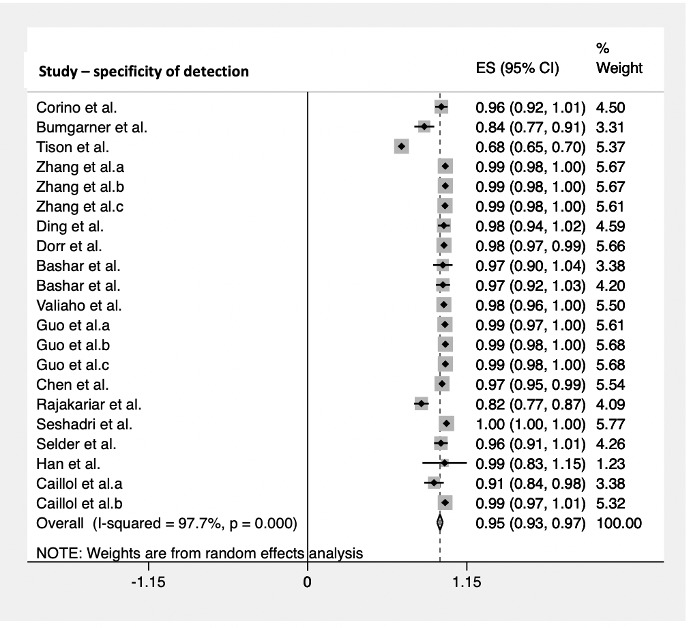
Pooled analysis for specificity of cardiac arrhythmia detection by smartwatches. Effect sizes are shown with 95% CIs. A random effects model was used. ES: effect sizes.

The diagnostic accuracy of the smartwatch in detecting cardiac arrhythmias was analyzed, reporting a pooled sensitivity of 100% (95% CI 0.99-1.00; Figure 2) in 17 studies with 5074 subjects and a pooled specificity of 95% (95% CI 0.93-0.97; Figure 3) in 16 studies with 5050 subjects. The sensitivity ranged from 25% (95% CI 0.14-0.36) to 100% (95% CI 1.00-1.00), whereas the specificity ranged from 68% (95% CI 0.65-0.70) to 100% (95% CI 1.00-1.00).

**Figure 4:**
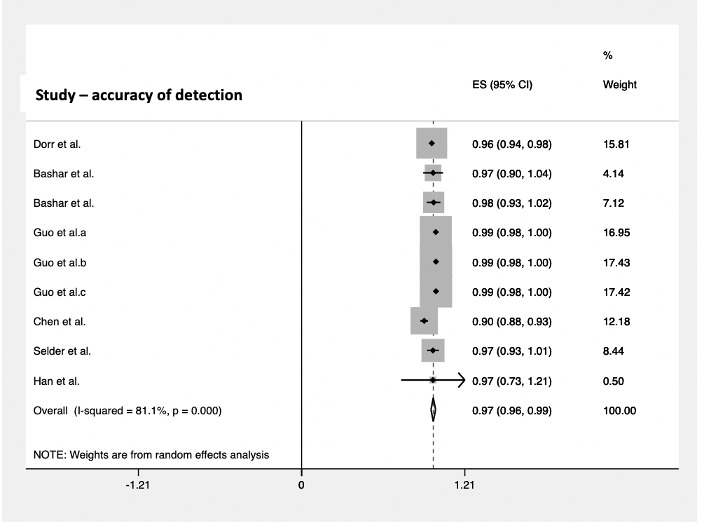
Pooled analysis for accuracy of cardiac arrhythmia detection by smartwatches. Effect sizes are shown with 95% CIs. A random effects model was used. ES: effect sizes.

Of the 18 studies, 7 (39%) reported data on accuracy. Among the 1769 subjects, the pooled accuracy for arrhythmia detection was 97% (95% CI 0.96-0.99; Figure 4).

### PPV and NPV Analysis

**Figure 5:**
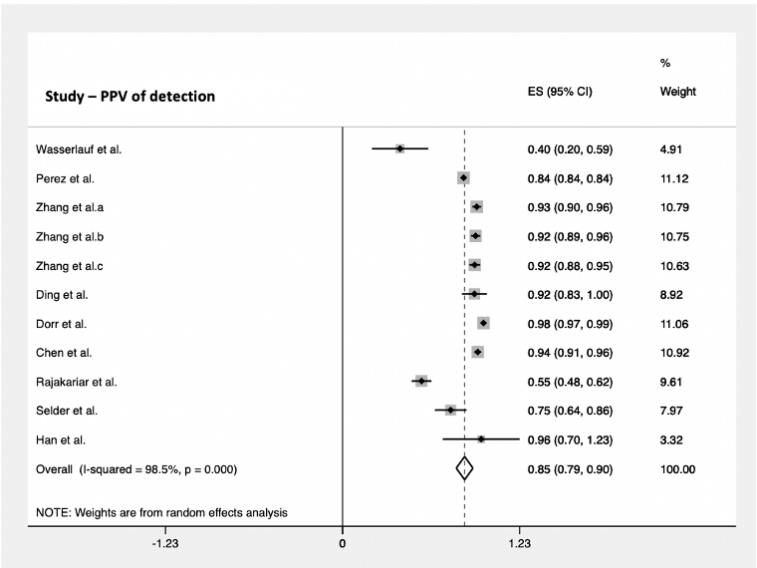
Pooled analysis for PPV of cardiac arrhythmia detection by smartwatches. Effect sizes are shown with 95% CIs. A random effects model was used. ES: effect sizes; PPV: positive predictive value.

**Figure 6:**
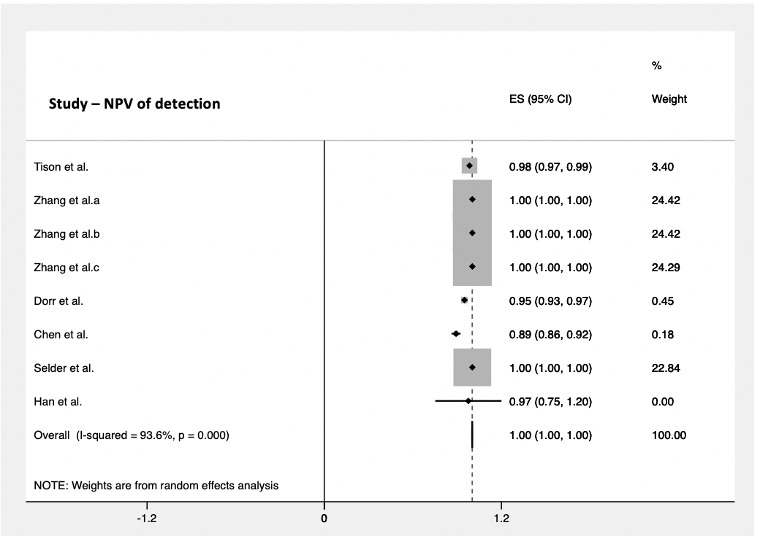
Pooled analysis for NPV of cardiac arrhythmia detection by smartwatches. Effect sizes are shown with 95% CIs. A random effects model was used. ES: effect sizes; NPV: negative predictive value.

The PPV for cardiac arrhythmia detection was assessed in 9 studies using a smartwatch. These included a total of 421,267 subjects and reported a PPV of 85% (95% CI 0.79-0.90; Figure 5). The pooled NPV was reported in 6 studies as 100% (95% CI 1.0-1.0; Figure 6), taking into consideration 3323 subjects.

### Heterogeneity of Studies

There was a high degree of variation between studies assessing cardiac arrhythmia detection using a smartwatch. The heterogeneity was statistically significant when all the studies were compared (*P*<.05). The lowest variation among studies was seen when reporting the accuracy of smart devices to detect arrhythmias (*I^2^*=81.1%), whereas heterogeneity was highest in studies when assessing PPV (*I^2^*=98.5%).

### Quality Assessment

The assessment of bias using the Quality Assessment of Diagnostic Accuracy Studies 2 tool for the included studies is highlighted in Multimedia Appendix 2 [14-31].

## Discussion

### Principal Findings

To the best of our knowledge, this systematic review and meta-analysis is the first to investigate the diagnostic accuracy of smartwatches for all cardiac arrhythmias. We have shown that the detection of cardiac arrhythmias using commercially available smartwatches is possible, with very high diagnostic accuracy. The overall sensitivity, specificity, and accuracy of these digital systems were 100%, 95%, and 97%, respectively. The pooled PPV and NPV for detecting cardiac arrhythmias were 85% and 100%, respectively. These values may offer clinicians a quantifiable appreciation for the use of smartwatches in a health care setting.

Although the aim of this study is to review the diagnostic accuracy of smartwatches in detecting cardiac arrhythmias, it is clear from the results that there are currently very few studies that assess the ability of PPG technology on smartwatches to detect non-AF arrhythmias.

### Smartwatches

**Figure 7:**
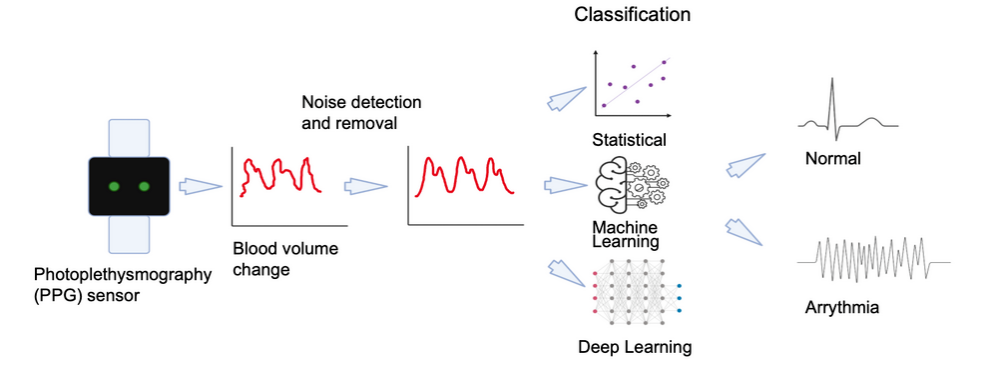
Overview of photoplethysmography sensor detection of arrhythmia. PPG: photoplethysmography.

A wide variety of smartwatches are commercially available, and this is reflected in the diverse range of smartwatches used in these studies (Table 2). These devices range from fitness trackers to more medically oriented watches with prices between US $40 and US $1700. Although all devices use PPG sensors (Figure 7), there is diversity in functionality beyond this point. Several smartwatches are capable of recording a single-lead ECG, and others, such as the Empatica E4, have electrodermal activity sensors capable of recording sympathetic nervous system activity. The Samsung Simband is unique within these studies in that it is the only device designed for developers and is not commercially available, allowing custom adaption of sensor inclusion. Of the studies included, only the Apple Smartwatch has Food and Drug Administration (FDA) clearance for its ECG tracking functionality.

**Table 2:** Characteristics of smartwatches used in included studies.

Smartwatch	Company	Country	Approximate price^a^, £ (US $)	Type	Photoplethysmography	Single-lead ECG^b^	Food and Drug Administration clearance ECG tracking	Electrodermal activity sensor
Apple Watch	Apple	USA	388 (531)	Watch	✓^c^	✓	✓	
Honor Watch	Honor	China	86 (117)	Watch	✓			
Huawei GT	Huawei	China	89 (122)	Watch	✓			
Gear S3	Samsung	South Korea	160 (219)	Watch	✓			
Simband	Samsung	South Korea	N/A^d^	Watch	✓	✓		✓
Honor Band	Honor	China	45 (61)	Fitness Band	✓			
Amazfit Healthband	Huami	China	33 (45)	Fitness Band	✓			
GearFit2	Samsung	South Korea	49 (67)	Fitness Band	✓			
Wavelet wristband	Biostrap or Wavelet Health	USA	180^e^ (246)	Wristband	✓			
Empatica E4	Empatica	USA	1227^f^ (1682)	Wristband	✓	✓		✓

^a^Pricing as per Amazon UK website on 22/04/2021.

^b^ECG: electrocardiogram.

^c^Included with smartwatch.

^d^N/A: not applicable or data not available.

^e^Pricing as per Biostrap shop on 22/04/2021.

^f^Pricing as per Empatica store on 22/04/2021.

### The Impact of Improving AF Detection

The incidence of AF increases annually with an increase in the prevalence of risk factors, such as advancing age, obesity, hypertension, and type 2 diabetes. The challenge with detection is the ability of AF to remain asymptomatic or intermittent before eventually revealing itself. This poses a huge economic burden, accounting for 1%-2% of health care expenditure [32]. A new technology that is promising for reducing or preventing AF-related morbidity, and in doing so, addressing this burden, is welcomed. Machine learning coupled with smartwatches provides the opportunity to detect asymptomatic arrhythmias in a timely manner, allowing appropriate management to be initiated early. A recent study showed that a trained deep neural network was able to outperform single cardiologists by accurately classifying a broad range of rhythm classes and distinguishing between artifacts and arrhythmias [33]. This method could reduce the rate of misdiagnosed rhythms by digital ECG machines and improve the efficiency of expert human ECG interpretation by accurately prioritizing the most urgent conditions.

The detection of cardiac arrhythmias using smartwatches has multiple functionalities. It can be used to diagnose an abnormal rhythm, for monitoring of an arrhythmia, for example, in those with known paroxysmal AF, or for screening. Current methods of AF detection are criticized for their periodic investigative approach, during which an irregular pulse may be absent [34-36]. Using smartwatches, users can diagnose an irregular pulse by placing a finger on their device at any point. Smartwatch devices that detect cardiac arrhythmias are a simple, noninvasive, and user-friendly alternative to current ECG monitoring tools, such as 24-hour Holter monitoring or implantable cardioverter defibrillators [37,38]. The novel devices provide users with prospective information in real time, with relatively high sensitivity and specificity, as shown in our study, and are cost-effective [39]. However, the adoption of this technology by clinicians and patients requires clinically meaningful results in a manner that is compatible with the workflow of clinicians. Therefore, an optimal strategy for their implementation must be in place.

### What Are the Next Steps?

Wearable devices for *wellness* are viewed as low-risk fitness monitors by the FDA, which does not apply the same stringent regulations as it would when considering medical devices. The FDA has introduced its Digital Health Precertification Program, in which companies are able to gain expedited clearance for ECG analysis and heart rate sensing software [40]. This process leads to companies producing technology that is confirmed to be *safe* but not necessarily of good quality because they have bypassed the conventional workflow for research discovery. Large clinical trials are lacking, and as a result, no expert consensus recommends screening for all occult AF [41].

Furthermore, there is insufficient evidence on the burden of smartwatch-detected AF, which would prompt further evaluation and treatment. Guidance on what the clinician is expected to do with an episode of AF detected by a smartwatch is lacking. We suggest that this should be a critical prerequisite before introducing a digital detection tool into the general population; otherwise, overdiagnosis and an expectant role of clinicians from the public to assess their device-detected condition will become an even bigger burden on the health care system. A recent study evaluating the clinical outcome of the Apple smartwatch concluded that false-positive screening results may lead to overutilization of the health care system [42]. Preparation for the problems that a new generation of smartwatch technology, which attempts to bridge the gap between disease and the health care system, brings is key.

With evolving technology in the field of health care applications, there is a move to a more personalized and *patient-centric* approach, where patients have an increasing number of tools at their disposal to assess risk and diagnose disease. Although frequent and active screening using a smartwatch is potentially feasible, few studies have examined the long-term adherence to this system. This user-involved measurement could too easily miss minimally symptomatic and brief paroxysms of arrhythmia. Long-term commitment and adherence from the user or the ability of continuous monitoring by the device is required for an accurate and worthwhile outcome.

### Limitations

There are many limitations to the studies in our review. At present, most studies have assessed the use of a PPG sensor and an accompanying algorithm to detect cardiac arrhythmias. However, they have not gone further to assess the use of such systems in health care. The largest study within our systematic review did not go beyond the participants’ self-reporting of an irregular pulse [20]. Several factors must be controlled to produce unbiased data that are clinically applicable. The published papers included observational and case-control studies, which did not evaluate the efficacy of smartwatch-based screening for clinical outcomes nor reflect real-life conditions [21,22]. Moreover, the sample sizes of some studies were small, with data sets of less than 50 in 5 of the papers [16,21,23,24,30]. One study had a very low sensitivity compared with others when assessing atrial flutter or tachycardia, which could likely be because of the small sample size for this group [31]. Finally, most studies were conducted in controlled research environments as opposed to a real-life setting, which may call into question the diagnostic accuracy of these smartwatches in an uncontrolled environment. Therefore, the interpretation of a sensitivity of 100%, effectively ruling out the presence of a cardiac arrhythmia with a negative result and the interpretation of an NPV of 100%, suggesting the return of no false negatives, should be interpreted with caution. The significant heterogeneity between studies is likely a result of different study settings, different patient group sizes, and different devices, based on personalized algorithms, having been used. Although the presence of this heterogeneity demands caution in interpreting our results, it also stresses the need for randomized controlled trials in this field using large data sets.

Many studies had a large proportion of data excluded because of insufficient PPG signal quality [14,18,22,24]. Some studies took place in settings where patients were supervised and provided instructions on the technique [22,27]. Thus, generalizing these findings to the *real world* could weaken the diagnostic accuracy. PPG technology recognizes the cardiac cycle by the pulsatile pattern of the change in light absorption, which reflects the volumetric alteration in the microvascular beds underneath the skin. With an accurate estimation, each episode of maximum reflected light absorption translates into an R wave. Although previous research has questioned the use of PPG sensors in darker skin, a recently published study showed no statistically significant differences in wearable heart rate measurement accuracy across skin tones [43]. However, a number of studies have shown that PPG sensors are less reliable at higher heart rates and during exercise [44,45]. As some studies in this review did not report the average heart rate of participants, it may add a level of bias to the results. In addition, PPG technology cannot detect myocardial ischemia or arrhythmias with a ventricular origin and therefore, at present, cannot completely replace 12-lead ECGs. Therefore, one must question whether the application of PPG-based sensors for cardiac arrhythmia detection is premature.

Finally, for smartwatch devices to be used as a screening tool for cardiac arrhythmias, such as AF detection, the value is highly dependent on disease prevalence. The estimated prevalence of AF in adults is between 2% and 4%. The prevalence increases with age, especially for those aged >65 years [46]. However, only 4.6% of smartwatch users in the United States are aged >65 years, and among those that are current smartwatch users, the prevalence of AF is low [47]. The studies in this review used estimated disease prevalence rates, which have been age adjusted, when assessing diagnostic accuracy values. Subjects were not limited to age groups, and as a result, some studies overestimated AF prevalence among smartwatch users [19,22]. This means that the PPV value of 85% in our review may be higher than expected for cardiac arrhythmia detection in smartwatch users. Either way, there is a high number of false positives, which leads to unnecessary anxiety among those in whom the device detects AF and may have the downstream consequences of inappropriate initiation of treatment in these patients. Treatment with anticoagulants can cause bleeding, which may be harmful. False positives may improve if the device is targeted to those most at risk of AF, but larger studies are needed to evaluate smartwatches as a tool for long-term AF screening in selected at-risk patient groups.

Regardless of the current studies, the future of health technology is undeniably advancing. Thus, measures should be taken early to ensure that such smartwatch technology supports ongoing national public health programs rather than having it run in parallel. Given the lack of recent success with the national NHS test and trace program in the United Kingdom, in which it fell short of its uptake aims when reaching contacts of people who tested positive for SARS-CoV-2 [48], the wider use of machine learning smartwatch technology should be considered in such circumstances. It may be more efficient and effective to integrate the need for health programs at the population level with existing devices. Governments should consider this, where applicable, in their decision-making processes.

### Conclusions

This systematic review and meta-analysis demonstrates the evolving field of digital disease detection and the increased role of machine learning in health care. The current diagnostic accuracy of smartwatch technology for the detection of cardiac arrhythmias is high. This shift signals a new direction in the field, allowing patients to play a greater role in disease diagnosis. However, before the use of these devices as a screening tool in health care is widely adopted, more studies are needed to clearly define the ideal population for the use of these systems, as well as to help form specific guidance on the conduct of device-detected disease. Consideration should also be placed for the wider use of smartwatch technology and similar digital tools in policy making decisions by health care departments in the future. Although the innovative drive of digital devices in health care will continue to gain momentum toward screening, the process of accurate evidence accrual and regulatory standards ready to accept their introduction is strongly needed.

## Appendix

Multimedia Appendix 1 Search strategy.

Multimedia Appendix 2 Quality assessment.

## Abbreviations

AF: atrial fibrillation
ECG: electrocardiogram
FDA: Food and Drug Administration
MeSH: Medical Subject Headings
NPV: negative predictive value
PPG: photoplethysmography
PPV: positive predictive value
PRISMA: Preferred Reporting Items for Systematic Reviews and Meta-Analyses
