# Supplementary material for: Diagnostic Accuracy of Smartwatches for the Detection of Cardiac Arrhythmia: Systematic Review and Meta-analysis
Source: J Med Internet Res. 2021 Aug 27;23(8):e28974. doi: 10.2196/28974 (PMC8433941; doi:10.2196/28974)
Supplement: Multimedia Appendix 1 [file jmir_v23i8e28974_app1.docx]

**Multimedia Appendix 1**

Medline / EMBASE

1. exp Arrhythmias, Cardiac/

2. atrial flutter

3. Atrial Fibrillation/

4. atrial fibrillation

5. irregular pulse*

6. irregular heart rhythm*

7. arrhythm*

8. 1 or 2 or 3 or 4 or 5 or 6 or 7

9. wearable electronic devices/ or fitness trackers/

10. smartwatch*

11. smartphone watch*

12. wristband*

13. wrist band*

14. 9 or 10 or 11 or 12 or 13

15. diagnosis, computer-assisted/ or image interpretation, computer-assisted/

16. detect*

17. diagnos* accuracy

18. diagnos*

19. 15 or 16 or 17 or 18

20. 8 and 14 and 19

The Cochrane Library

#1 MeSH descriptor: [Arrhythmias, Cardiac] explode all trees

#2 atrial flutter

#3 atrial fibrillation

#4 irregular pulse*

#5 irregular heart r*

#6 arrhythm*

#7 #1 or #2 or #3 or #4 or #5 or #6

#8 MeSH descriptor: [Fitness Trackers] explode all trees

#9 smartwatch*

#10 wearable electronic device*

#11 smartphone watch*

#12 wristband*

#13 wrist band*

#14 #8 or #9 or #10 or #11 or #12 or #13

#15 MeSH descriptor: [Diagnosis, Computer-Assisted] this term only

#16 detect*

#17 diagnos* accuracy

#18 diagnos*

#19 #15 or #16 or #17 or #18

#16 #7 AND #14 AND #19

**Figure S1.** Search Strategy
