# Supplementary material for: Diagnostic Accuracy of Smartwatches for the Detection of Cardiac Arrhythmia: Systematic Review and Meta-analysis
Source: J Med Internet Res. 2021 Aug 27;23(8):e28974. doi: 10.2196/28974 (PMC8433941; doi:10.2196/28974)
Supplement: Multimedia Appendix 2 [file jmir_v23i8e28974_app2.docx]

**Multimedia Appendix 2**

| **Study** | **Risk of bias** | | | | **Applicability concerns** | | |
| --- | --- | --- | --- | --- | --- | --- | --- |
|  | **Patient selection** | **Index test** | **Reference standard** | **Flow and timing** | **Patient selection** | **Index test** | **Reference standard** |
| Wasserlauf et al. 2019 | ? | ☺ | ☺ | ☺ | ☺ | ☺ | ☺ |
| Perez et al. 2019 | ☺ | ☺ | ☺ | ☺ | ? | ? | ☺ |
| Bumgarner et al. 2018 | ☺ | ☺ | ☺ | ? | ☺ | ☺ | ☺ |
| Rajakariar et al. 2020 | ☺ | ☺ | ☺ | ? | ☹ | ☺ | ? |
| Ding et al. 2019 | ☹ | ☺ | ☹ | ☹ | ☺ | ? | ? |
| Dorr et al. 2019 | ☹ | ☺ | ☺ | ? | ? | ☺ | ? |
| Bashar et al. 2019 | ☹ | ? | ☺ | ☺ | ☹ | ? | ☺ |
| Bashar et al. 2019 | ☹ | ☺ | ☺ | ? | ☹ | ☺ | ☺ |
| Chen et al. 2020 | ☹ | ☺ | ☺ | ? | ? | ☺ | ☹ |
| Corino et al. 2017 | ☺ | ☺ | ☹ | ☺ | ☺ | ? | ☹ |
| Tison et al. 2018 | ☹ | ☺ | ☺ | ☺ | ☺ | ☺ | ☺ |
| Zhang et al. 2019 | ? | ☺ | ? | ☺ | ☺ | ☺ | ☺ |
| Valiaho et al. 2019 | ☺ | ☺ | ☺ | ☺ | ☺ | ☹ | ☹ |
| Guo et al. 2019 | ☹ | ☺ | ☺ | ☺ | ☺ | ☺ | ☺ |
| Sheshadri et al. 2020 | ☺ | ☺ | ☺ | ☺ | ☹ | ☹ | ☺ |
| Selder et al. 2020 | ☺ | ☺ | ☹ | ? | ☺ | ☺ | ☹ |
| Han et al. 2020 | ? | ☺ | ☺ | ☺ | ☹ | ☺ | ☺ |
| Caillol et al. 2021 | ? | ☺ | ☺ | ? | ☺ | ☺ | ☺ |

**Figure S2.** Assessment of bias for each included study in seven domains according to the QUADAS-2 tool. ☺ = low risk of bias; ☹ = high risk of bias; ? = unclear risk of bias.
